# Stereotactic radiosurgery combined with nivolumab or Ipilimumab for patients with melanoma brain metastases: evaluation of brain control and toxicity

**DOI:** 10.1186/s40425-019-0588-y

**Published:** 2019-04-11

**Authors:** Giuseppe Minniti, Dimitri Anzellini, Chiara Reverberi, Gian Carlo Antonini Cappellini, Luca Marchetti, Federico Bianciardi, Alessandro Bozzao, Mattia Osti, Pier Carlo Gentile, Vincenzo Esposito

**Affiliations:** 1 Radiation Oncology Unit, UPMC Hillman Cancer Center|, San Pietro Hospital FBF, 00189 Rome, Italy; 2 Radiation Oncology Unit, Sant’ Andrea Hospital, University Sapienza, 00100 Rome, Italy; 3 IV Oncology Division, Istituto Dermopatico dell’Immacolata IRCCS, Rome, Italy; 4 Neuroradiology Unit, Sant’ Andrea Hospital, University Sapienza, 00189 Rome, Italy; 5 IRCCS Neuromed, 86077 Pozzilli (IS), Italy

**Keywords:** stereotactic radiosurgery, melanoma brain metastases, fractionated stereotactic radiosurgery, checkpoint inhibitors, immunotherapy

## Abstract

**Purpose:**

To investigate the efficacy and safety of concurrent stereotactic radiosurgery (SRS) and ipilimumab or nivolumab in patients with untreated melanoma brain metastases.

**Patients and Methods:**

Eighty consecutive patients with 326 melanoma brain metastases receiving SRS in combination with ipilimumab or nivolumab were identified from an institutional database and retrospectively evaluated. Patients started systemic treatment with intravenous nivolumab or ipilimumab within one week of receiving SRS. Nivolumab was given at doses of 3 mg/kg every two weeks. Ipilimumab was administered up to four doses of 10 mg/kg, one every 3 weeks, then patients had a maintenance dose of 10 mg/kg every 12 weeks, until disease progression or inacceptable toxicity. Primary endpoint of the study was intracranial progression-free survival (PFS). Secondary endpoints were extracranial PFS, overall survival (OS), and neurological toxicity.

**Results:**

Eighty patients were analyzed. Forty-five patients received SRS and ipilimumab, and 35 patients received SRS and nivolumab. With a median follow-up of 15 months, the 6-month and 12-month intracranial PFS rates were 69% (95%CI,54–87%) and 42% (95%CI,24–65%) for patients receiving SRS and nivolumab and 48% (95%CI,34–64%) and 17% (95%CI,5–31%) for those treated with SRS and ipilimumab (p = 0.02), respectively. Extracranial PFS and OS were 37 and 78% in SRS and nivolumab group, respectively, and 17 and 68% in SRS and ipilimumab group, respectively, at 12 months. Sub-group analysis showed significantly better intracranial PFS for patients receiving multi-fraction SRS (3 × 9 Gy) compared to single-fraction SRS (70% versus 46% at 6 months, *p* = 0.01), especially in combination with nivolumab. Grade 3 treatment-related adverse events occurred in 11 (24%) patients treated with SRS and ipilimumab and 6 (17%) patients who received SRS and nivolumab. Radiation-induced brain necrosis (RN) occurred in 15% of patients.

**Conclusions:**

Concurrent SRS and ipilimumab or nivolumab show meaningful intracranial activity in patients with either asymptomatic and symptomatic melanoma brain metastases, although a subset of patients may develop symptomatic RN. The combination of nivolumab with SRS is associated with better intracranial control.

**Electronic supplementary material:**

The online version of this article (10.1186/s40425-019-0588-y) contains supplementary material, which is available to authorized users.

## Introduction

Brain metastases are a common and devastating complication of cancer affecting 25% of patients with advanced melanoma [1]; for these patients, systemic therapy and local treatments, including surgical resection, whole brain radiation therapy (WBRT) and stereotactic radiosurgery (SRS) have been the most common therapeutic options.

Systemic chemotherapy has been widely used in the past for patients with melanoma brain metastases, although it has shown a limited activity. Local control (LC) has improved with the use of surgery and radiotherapy, given alone or in combination [1]. Historically, WBRT has been the cornerstone for treatment of multiple brain metastases, but its use has been progressively replaced by SRS; its efficacy in patients with a limited number of lesions, usually 1-4, has been demonstrated in randomized trials [2, 3], although LC in melanoma patients is inferior than that reported for other histologies, especially when considering large tumors [4–7].

In the last few years systemic therapies have evolved; targeted therapies with BRAF and MEK inhibitors and immunotherapy with PD-1/PD-L1 or CTLA-4 checkpoint inhibitors, given alone or in combination, have significantly improved survival in patients with melanoma brain metastases [8–12]. In a randomized phase 2 study of 60 patients with melanoma brain metastases receiving combined nivolumab and ipilimumab or nivolumab alone, Long et al. [11] showed an intracranial response of 46% and 20%, respectively; with a median follow-up of 17 months, 6-month intracranial progression-free survival (PFS) and overall survival (OS) rates were 35% and 68% in patients receiving nivolumab, and 53% and 78% in those receiving nivolumab and ipilimumab. In another phase 2 study of 94 patients with melanoma brain metastases treated with combined nivolumab and ipilimumab, Tawbi et al. [12] observed an intracranial objective response of 55% lasting at least 6 months, with PFS rates of 70.4% and OS rates of 59.5% at 9 months.

In patients with melanoma brain metastases, stereotactic radiosurgery SRS in combination with checkpoint inhibitors may be associated with improved efficacy over SRS alone [13]; however, timing and sequence of combined SRS and checkpoint inhibitors are highly variable among different studies, and the efficacy and toxicity of treatment remains to be defined. In our study we have evaluated the efficacy and safety of SRS combined with nivolumab or ipilimumab in patients with untreated melanoma brain metastases.

## Patients and Methods

Between September 2012 and December 2017, 112 consecutive patients ≥18 years old receiving combined SRS and ipilimumab or nivolumab for one to ten melanoma brain metastases were retrospectively evaluated. In general, patients with lesions up to 2.5 cm in size were treated with single-fraction SRS, while larger lesions located near or in eloquent areas (i.e., motor, somatosensory, speech, visual cortices, basal ganglia, thalamus, and the brainstem) received multi-fraction SRS to minimize potential increased risk of late radiation-induced brain necrosis (RN).

All radiographic, surgical, and pathological information were drawn from a prospectively maintained database of patients with brain tumors treated at Sant’ Andrea Hospital and UPMC Hillman Cancer Center San Pietro Hospital. Thirty-two patients were excluded due to insufficient clinical information, previous use of anti-PD-1/PD-L1, brain surgery or radiation. Previous adjuvant therapies, including ipilimumab or BRAF/MEK inhibitors, were allowed. A total of 80 patients with 326 brain metastases were finally analyzed. All patients provided written consent to the treatment. Local Institutional Review Boards at Sant’ Andrea and San Pietro Hospitals approved this retrospective study.

All lesions were treated with LINAC-based SRS (TrueBeam STx and Clinac 2100 linear accelerators, Varian Medical System) using a commercial stereotactic mask fixation system (BrainLab). Target volumes were contoured on thin-slice (1-mm) gadolinium-enhanced T1-weighted axial MRI sequences fused with planning computed tomography (CT) scans. The gross tumor volume (GTV) was delineated as the contrast-enhancing tumor demonstrated on MRI scans. The planning tumor volume (PTV) was generated giving a geometric expansion to GTV of 0.5-1 mm. In patients undergoing single-fraction SRS, doses were 22 Gy for lesions <2 cm and 18 Gy for those between 2 and 3 cm in size. For lesions treated with multi-fraction SRS, a dose of 27 Gy in 3 fractions was delivered on consecutive days. The choice of 3x9 Gy was made on the basis of radiobiological consideration and previous clinical experiences [14, 15]. According to the linear quadratic model for the estimation of dose-effect relationship adjusted for high doses [14], the biological effective dose (BED) of 27 Gy in 3 fractions is 40 Gy that corresponds to a single dose of about 22 Gy, assuming an α/β of 12 Gy (BED_12_) for brain metastases. Doses were generally prescribed to the 80% isodose line and delivered using 4-7 noncoplanar dynamic or volumetric arcs. Cone-beam CT and ExacTrac*®* image-guided systems were used to ensure accurate patient positioning. In patients with significant or symptomatic perilesional edema, a maximum dose of 4 mg dexamethasone per day was allowed at the time of SRS, then maintained for 3-7 day.

Concurrent systemic treatment consisted of - intravenous nivolumab administered at doses of 3 mg/kg every two weeks, or - intravenous ipilimumab up to four doses of 10 mg/kg, one in every 3 weeks, then a maintenance dose of 10 mg/kg every 12 weeks, until disease progression or inacceptable toxicity. Based on preclinical evidences that early release of tumor antigens and activation of tumor-specific T cells following SRS may enhance the effects of immunotherapy [16, 17], ipilimumab and nivolumab were generally administered 48-72 hours before receiving SRS. The choice of treatment was mainly based on the availability of checkpoint inhibitors for clinical standard practice in Italy. For patients with metastatic melanoma, the Italian Medicine Agency (AIFA) approved ipilimumab in February 2013 and nivolumab in March 2016. This means that ipilimumab was the only choice between 2013 and 2016, while nivolumab has been used more frequently since 2016 in patients with either BRAF wild-type melanoma or who had previously received BRAF/MEK inhibitors and ipilimumab. Salvage therapies at progression were chosen by the treating physicians; selected patients with clinical benefits from systemic treatments were allowed to continue nivolumab beyond progression.

Patients were clinically examined approximately at 2-6 weeks intervals. At each visit, neurological status and severity of complications were recorded according to the Common Terminology Criteria for Adverse Events 4.0. MRI was made every 2 months in the first year after the treatment, and subsequently every 2-3 months or as appropriate. For brain metastases measuring ≥5 mm, intracranial complete response (CR), partial response (PR), stable disease (SD), and progressive disease (PD) were determined by MRI according to the modified response evaluation criteria in solid tumors criteria (mRECIST v1.1.) [18], with tumor measurements and reporting of scans carried out by the same neuroradiologist (A.B.). Pseudoprogression was defined as transient increased contrast enhancement and edema occurring few months from SRS which resolved or stabilized during subsequent follow-up. Extracranial response was assessed according to RECIST v1.1. [19]. Diagnosis of tumor progression or RN were determined on the basis of histological findings (for patients who underwent surgical resection) or with imaging using MRI and 3,4-dihydroxy-6-(18) F-fluoro-l-phenylalanine-(F-DOPA)-PET-CT, as previously reported [20].

## Outcomes and data analysis

Primary endpoint was intracranial PFS. Secondary endpoints were extracranial PFS, OS, and neurological toxicity. Time-to-event analysis were estimated using the Kaplan-Meier method from the date of SRS. Chi-square and non-parametric Mann-Whitney tests were used to examine between-group covariate differences, and the Cox proportional hazards model was employed for univariate and multivariate analysis to assess the effects of clinical/treatment variables on outcomes. Variables included in the univariate analysis were age at diagnosis, gender, KPS score, previous systemic treatments, number of metastases, extracranial disease status, diagnosis-specific graded prognostic assessment (DS-GPA) score [21], type of SRS, total tumor volume, GTV, and PTV. Variables at significance levels of *p*<0.05 were included in multivariate analysis. Standard softwares were used for statistical analysis (SAS software, version 9.3; XLSTAT).

## Results

### Patient characteristics

A total of 80 consecutive patients with 326 untreated melanoma brain metastases who received SRS for 1-10 lesions combined with ipilimumab or nivolumab were analyzed. Patient characteristics are shown in Table 1. Forty-five patients received concurrently SRS and ipilimumab, and 35 patients SRS and nivolumab with a median interval between infusion and SRS of 3 days (range 2-7 days). There were no significant differences between groups in terms of gender, age, number of metastases, KPS scores, irradiated volumes, DS-GPA, and type of SRS (single-fraction or multi-fraction SRS). Forty-one patients received multi-fraction SRS for at least one metastasis. Fifty-six patients with extracranial metastases had one or two lines of systemic therapy prior to SRS; among them, twenty-eight patients with BRAF-mutated tumors were previously treated with BRAF/MEK inhibitors, and 7 patients received ipilimumab.

**Table 1:** Patient characteristics and treatment parameters

Variable	SRS and ipilimumab	SRS and nivolumab	
	*N* = 45	*N* = 35	p
*Sex (F/M)*	17/28	14/21	1.0
*Age (years)*			
median	54	56	0.2
range	23–78	26–80	
*KPS*			0.8
median	80	80	
60–70	13	9	
80–100	32	26	
*BRAF mutation*			0.8
present	15	13	
absent	30	22	
undetermined			
*Extracranial disease*			0.7
present	34	25	
absent	11	10	
*Number of metastases*			0.4
single	8	9	
multiple	37	26	
*DS-GPA*			0.3
0–1	9	6	
1.5–2.5	22	16	
3–4	14	13	
*Type of SRS*			0.76
Single-fraction SRS	153	132	
Fractionated SRS	22	19	
Size of metastases			0.8
< 2 cm	99	84	
2–3 cm	46	38	
≥ 3 cm	30	29	
*Total tumor volume (cm* ^*3*^ *)*			0.1
median	7.4	9.2	
range	0.5–33.1	0.7–33	
*GTV (cm* ^*3*^ *)*			0.6
median	1.12	1.2	
range	0.05–27.9	0.4–31.2	
*PTV (cm* ^*3*^ *)*			0.3
median	1.71	1.83	
range	0.1–39.1	0.09–42.6	
*Conformity index* ^*a*^			0.5
median	1.43	1.41	
range	1.10–1.91	1.12–1.85	

*SRS* stereotactic radiosurgery, *KPS* Karnofsky Performance Status

*DS-GPA* Diagnosis-Specific Graded Prognostic Factors, *GTV* Gross Target Volume

*PTV* Planning Target Volume, ^a^calculated as prescribed isodose volume/tumor volume

encompassed by the prescription isodose volume

For progressive disease, 27 patients received subsequent systemic therapy, including chemotherapy (SRS and ipilimumab, 8; SRS and nivolumab, 4), BRAF/MEK inhibitors (SRS and nivolumab, 3), and checkpoint inhibitors (SRS and ipilimumab, 8; SRS and nivolumab, 4). Eight patients who progressed on ipilimumab received nivolumab or pembrolizumab, whereas 7 patients in SRS and nivolumab group received trametinib and dabrafenib or combined ipilimumab and fotemustine as salvage therapies. In addition, 7 asymptomatic patients with good performance status continued nivolumab administration beyond intracranial progression. At the time of intracranial progression, local salvage therapies included surgery (*n* = 9), SRS (*n* = 29), and WBRT (*n* = 8). Dexamethasone up to 4 mg per day for more than 2 weeks was given in 37 patients at the time of SRS (*n* = 23) or to manage toxicity (*n* = 14). At the time of analysis (July 2018), 17 patients were still undergoing treatment; 51 (64%) had died.

### Progression-free survival and survival

**Fig. 1:**
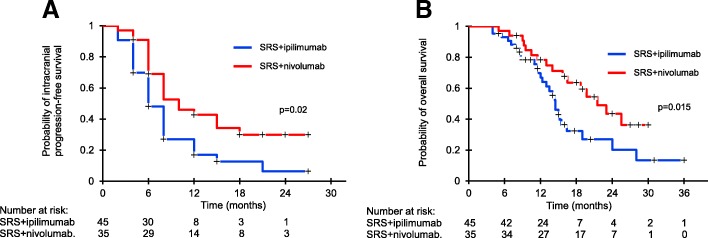
Kaplan-Meier analysis of overall survival (OS, **a**) and intracranial progression-free survival (PFS, **b**) for patients receiving concurrent SRS and ipilimumab (blue line) or nivolumab (red line). OS and intracranial PFS were significantly better in SRS and nivolumab group

With a median follow-up of 15 months, 32 (71%) out of 45 patients in SRS and ipilimumab group, and 20 (57%) out of 35 patients in SRS and nivolumab group had an intracranial progression event, with a median intracranial PFS of 6 and 10 months (*p* = 0.02), respectively. The 6-month and 12-month intracranial PFS rates were 69% (95%CI, 54-87%) and 42% (95%CI, 24-65%) in SRS and nivolumab group and 48% (95%CI, 34-64%) and 17% (95%CI, 5-31%) in SRS and ipilimumab group (*p* = 0.02), respectively, (Fig. 1a). Median OS was 22.0 months in SRS and nivolumab group and 14.7 months in SRS and ipilimumab group (*p* = 0.015) (Fig. 1b); respective 12-month and 24-month survival probabilities were 78% (95%CI, 63-95%) and 42% (95%CI, 26-63%), and 68% (95%CI, 51-89%) and 20% (95%CI, 5-36%). Twenty-three patients succumbed to their intracranial disease (SRS and ipilimumab, 15; SRS and nivolumab, 8) and 28 patients died of progressive extracranial disease (SRS and ipilimumab, 17; SRS and nivolumab, 11).

**Fig. 2:**
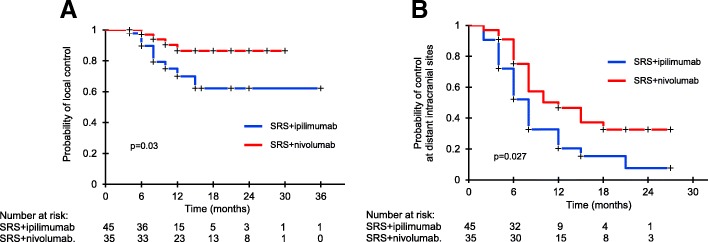
Kaplan-Meier analysis of local control (LC, **a**) and distant brain control (DBC, **b**) after concurrent SRS and ipilimumab (blue line) or nivolumab (red line). LC and DBC were significantly better in SRS and nivolumab group

Analysis of LC and distant brain control (DBC) showed significant differences by groups (Fig. 2). Four patients who received SRS and nivolumab and 10 who were treated with SRS and ipilimumab had local failure; 6-month and 12-month LC rates were 96% (95%CI, 87-100%) and 85% (95%CI, 75-95%) in SRS and nivolumab group, respectively, and 90% (95%CI, 81-99%) and 70% (95%CI, 59-81%) in SRS and ipilimumab group, respectively (*p* = 0.03). With a median time of 4 months, CR and PR occurred in 41% and 35% of patients receiving SRS and nivolumab, and 23% and 37% in those receiving SRS and ipilimumab, yielding to intracranial objective response rates of 76% and 60%. LC was similar for symptomatic and asymptomatic lesions. DBC rates were significantly different; 75% (95%CI, 59-93%) and 46% (95%CI, 29-65%) in SRS and nivolumab group and 52% (95%CI, 34-69%) and 20% (95%CI, 6-35%) in SRS and ipilimumab group at 6 and 12 months, respectively (*p* = 0.027).

The 6-month and 12-month extracranial PFS rates were 57% and 37% in SRS and nivolumab group and 42% and 17% in SRS and ipilimumab group (*p* = 0.03), respectively; global PFS rates were 53% and 36% and 34% and 17% (*p* = 0.02), (Additional file 1: Figure S1). The majority of patients had concurrent intracranial and extracranial progression; intracranial progression alone occurred in 5 patients receiving SRS and ipilimumab and 2 patients undergoing SRS and nivolumab.

### Analysis of prognostic factors

**Fig. 3:**
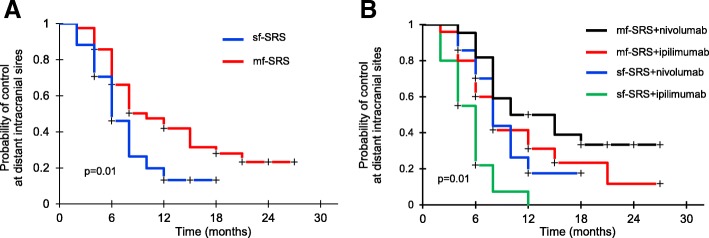
Kaplan-Meier analysis of intracranial progression-free survival (PFS) after single-fraction radiosurgery (sf-SRS, blue line) or multi-fraction SRS (mf-SRS, red line) in combination with ipilimumab or nivolumab. Patients receiving mf-SRS had significantly better intracranial PFS than those treated with sf-SRS (**a**); differences in PFS were seen in both ipilimumab and nivolumab groups (**b**)

For the whole population, multivariate analysis showed that SRS and nivolumab treatment, multi-fraction SRS, absent extracranial disease, and KPS >70 were significant indices of prolonged OS (Table 2). According to DS-GPA score, median OS was 6.8, 14.2 and 29.0 months for patients with scores of 0-1, 1-2.5, and 3-4 (*p <* 0.001), respectively. Patients who had received BRAF and MEK inhibitors treatment prior to the study showed a trend toward worse survival (*p* = 0.07): for this group, 12-month and 24-month OS rates were 46% and 16%, respectively. Multi-fraction SRS was associated with better intracranial PFS; 6-month and 12-month rates were 70% and 40% for patients receiving multi-fraction SRS and 46% and 10% for those undergoing single-fraction SRS (*p* = 0.01), respectively (Fig. 3). Groups sub-analysis showed that patients receiving multi-fraction SRS and nivolumab had better intracranial PFS (Fig. 3b). The use of steroids showed a trend toward worse OS (HR 1.74, 95%CI, 0.94-2.2; *p* = 0.057) and intracranial PFS (HR1.97, 95%CI, 0.91-2.4; *p* = 0.068) (Additional file 2: Figure S2). Aside from combined SRS and nivolumab, no other factors, including tumor size, irradiated volumes, number of lesions, and SRS type were predictor of LC. Concurrent nivolumab and SRS type resulted in better extracranial PFS rates; 6-month and 12-month rates were 61% and 40% in patients receiving multi-fraction SRS and 47% and 18% in those receiving single-fraction SRS, respectively (*p* = 0.03).

**Table 2:** Independent favorable prognostic factor for intracranial PFS* and OS

Outcome	Variable	Hazard ratio	95% CI	P
Intracranial PFS	SRS and nivolumab	0.54	0.32–0.92	0.038
	fmulti-fraction SRS	0.48	0.28–0.87	0.015
OS	SRS and nivolumab	0.51	0.28–0.81	0.019
	multi-fraction SRS	0.54	0.33–0.96	0.043
	KPS >70	0.34	0.23–0.78	0.010
	absent extracranial disease	0.50	0.29–0.81	0.018

Abbreviations: OS, overall survival; PFS, progression-free survival;HR, hazard ratio; CI, confidence interval; *Variables with a significance of *p* < 0.05 at univariate analysis were included in the multivariate analysis. The following variables were evaluated: age, gender, Karnofsly Perforance Status (KPS) score, histology, extracranial disease status, systemic therapy, number of metastases, time to brain metasases development, conformity index, and irradiated volumes

### Toxicity

A clinical neurological improvement after SRS occurred in 15 (63%) out of 22 patients with pre-existing neurological symptoms. Adverse events were recorded in 66.6% of patients having SRS and ipilimumab and 57% of those receiving SRS and nivolumab, with grade 3 events observed in 11 (24%) and 6 (17%) patients, respectively (Table 3). CNS-related grade 3 events were represented by headache (***n*** = 3), seizure (***n*** = 3), and brain edema (***n*** = 4). Intracranial hemorrhage was seen in 5 patients, being symptomatic in two (grade 3). Ipilimumab was discontinued in 5 patients and nivolumab in 3 patients. The most common extracranial grade 3 events were diarrhoea (***n*** = 4), increased serum aspartate aminotransferase (***n*** = 3), and rash [2]**.**

**Table 3:** Adverse events

	SRS and Ipilimumab (*n* = 45)		SRS and Nivolumab (*n* = 35)	
	Grade 1 or 2	Grade 3	Grade 1 or 2	Grade 3
Number of patients with at least an adverse event^a^	31 (68%)	11 (24%)	20 (57%)	6 (17%)
Event				
Diarrhoea	11 (24%)	3 (7%)	5 (14%)	1 (3%)
Nausea or vomiting	8 (18%)	1 (2%)	4 (12%)	1 (3%)
Constipation	5 (11%)	0	2 (6%)	0
Increased AST and/or ALT levels	4 (9%)	2 (4%)	4 (12%)	2 (6%)
Fatigue	12 (27%)	3 (7%)	6 (18%)	2 (6%)
Endocrine immune disorders	3 (7%)	0	2 (6%)	0
Rash/Pruritus	10 (22%)	1 (2%)	6 (18%)	1 (3%)
Arthralgia	5 (11%)	0	3 (9%)	0
Muscle weakness right or left sided	3 (7%)	1 (2%)	2 (6%)	1 (3%)
CNS event				
Headache	8 (18%)	2 (4%)	4 (12%)	1 (3%)
Hemorrhage	3 (7%)	1 (2%)	2 (6%)	1 (3%)
Seizure	3 (7%)	2 (4%)	2 (6%)	1 (3%)
Diziness	4 (9%)	0	2 (6%)	0
Brain necrosis	13 (29%)	5 (11%)	7 (20%)	3 (9%)
Discontinuation of treatment	5		3	

^a^Treatment-related adverse events of any grade occurring in at least 5% of patiens in either cohorts. Some patients had more than one event. No grade 4 events were reported in both cohorts

The risk of radiation-induced brain necrosis (RN) was evaluated by MRI/F-DOPA PET-CT studies. With a median time of 8 weeks (range 4-16 weeks), an early enlargement of irradiated lesions was recorded in 19 patients (43 lesions). Grade 3 neurological deficits related to imaging changes developed in 6 patients requiring medical therapy (Table 3). On subsequent imaging, tumor volumes decreased (***n*** = 19) or disappeared (***n*** = 21) in 13 patients at a median time of 8 weeks, confirming the diagnosis of pseudoprogression, and continued to enlarge in 3 patients who were recorded as having intracranial progression. Imaging criteria suggestive of RN were observed in further 12/80 patients (35/326 lesions) who were followed up for at least 6 months with MRI and DOPA PET-CT scans; SRS and nivolumab, 5/35; SRS and ipilimumab, 7/45). In 5 patients who underwent surgery, diagnosis of RN was confirmed by histology. The estimated 12-month incidence of RN was 25% in SRS and ipilimumab group and 17% in SRS and nivolumab group (*p* = 0.15); RN was symptomatic in 12 patients (SRS and ipilimumab, *n* = 7; SRS and nivolumab, *n* = 5), requiring surgery or long-term medical treatment. Grade 2 or 3 neurotoxicity, including motor deficits (*n* = 7), neurocognitive deficits (*n* = 3), seizure (*n* = 2), and speech deficits (*n* = 1), occurred in 7 and 2 patients, respectively.

No factors were independent predictors of RN, including tumor size, total tumor volume, GTV, PTV, and type of SRS; however, the median GTV was higher for symptomatic necrotic lesions (7.3 vs 2.7 ml; *p* = 0.003). The risk was similar after single-fraction SRS or multi-fraction SRS, even though the median GTV was significantly higher for lesions treated with multi-fraction SRS (11.7 vs 1.8 ml; *p* = 0.0001); for lesions > 2.0 cm in size, the 12-month estimated risk of RN was 28% and 16% after single-fraction SRS and multi-fraction SRS (*p* = 0.07), respectively.

## Discussion

Results of this study show that SRS concurrently to nivolumab or ipilimumab has a meaningful intracranial efficacy in patients with either asymptomatic or symptomatic untreated melanoma brain metastases. The 6-month and 12-month intracranial PFS rates were 69% and 42% for patients undergoing SRS and nivolumab and 48% and 17% for those receiving SRS and ipilimumab (*p* = 0.02), respectively. Combined SRS and nivolumab was associated with significantly longer LC and DBC; local failure rates decreased from 10% to 4% and from 30% to 14%, and DBF rates from 48% to 25% and from 80% to 54% at 6 months and 12 months, respectively. Similarly, extracranial PFS and OS were significantly better with SRS and nivolumab, with more than 40% of patients expected to be alive at 2 years.

Our findings are consistent with results from recent retrospective series on the efficacy of combining SRS with checkpoint inhibitors (Table 4) [22–32]. In a series of 96 patients with 314 melanoma brain metastases who had SRS within 3 months from receiving different systemic therapies, Ahmed et al. [24] observed 12-month DBC rates of 38% and 21% after SRS and nivolumab or ipilimumab, respectively, and improved survival compared to conventional chemotherapy. In another series of 46 patients with a total of 113 melanoma brain metastases, Kiess et al. [23] reported an estimated 12-month LC, DBC and OS rates of about 87%, 37% and 60%, respectively, in patients receiving SRS before or concurrently to ipilimumab. A similar efficacy of SRS and pembrolizumab has been reported in other few studies [25, 31–33]. With regard to the treatment sequencing, published results suggest that SRS and checkpoint inhibitors given concurrently, typically within 4 weeks of SRS, are associated with improved intracranial control and survival compared to nonconcurrent therapy [23, 25, 29, 31, 34] or SRS given alone [22, 26], with no significantly increased neurotoxicity. Overall, our results provide further evidence supporting the efficacy of concurrent immunotherapy and SRS for melanoma brain metastases, even in patients with symptomatic and large lesions.

**Table 4:** Selected studies assessing the efficacy and toxicity of SRS and immunotherapy for the treatment of melanoma brain metastases

Authors	Patients (n)	Treatment	Median survival (months)	Brain control	Neurotoxicity	Brain necrosis (% of patients)
Knisely et al., 2012 [22]	16	Ipi after SRS	21.3	NR	NR	NR
	11	Ipi before SRS	19.8	NR	NR	NR
	50	SRS alone	4.9	NR	NR	NR
*Kiess* et al.*, 2015* [23]	15	Concurrent SRS and Ipi (within 1 month)	1-year 65%	1-year LC 100%	Grade 2, 33% Grade 3, 26%	Early and late RN 50% of patients treated during or before Ipi and 13% of patients treated after Ipi.
	19	Nonconcurrent, SRS before Ipi (median 3 months)	1-year 56%	1-year LC 87%	Grade 2, 10% Grade 3, 6% Grade 4, 3%	
	12	Nonconcurrent, SRS after Ipi (median 2 months)	1-year 40%	1-year LC 89%		
*Ahmed* et al.*, 2016* [24]	26	SRS/SRT and Nivo	78% (1-year 55%)	6-month and 1-year DBC 61 and 38% 6-month and 1-year LC 89 and 82%	Grade 2, 37%	27%
*Qian* et al.*, 2016* [25]	33	SRS and concurrent IPI (1) or Pembro (14)	19.1	NR	NR	NR
	42	Nonconcurrent SRS and Ipi (35) or Pembro (7)	9	NR	NR	NR
Choong et al., 2017 [26]	28	Concurrent SRS and Ipi (within 6 weeks)	7.5 (1-year 40%)	7.5 months	NR	0%
	11	Concurrent SRS and Nivo (within 6 weeks)	20.4 (1-year 78%)	12.7 months	NR	18%
Cohen-Inbar et al., 2017 [27]	32	Ipi before or during SRS	1-year 59.4%	1-year LC and DBF 54.4 and 15.8%	NR	31%
	14	Ipi after SRS	1-year 33%	1-year LC and DBF 16.5 and 26.8%	NR	7%
Gaudi-Marqueste et al., 2017 [28]	21	SRS before Ipi (21), Nivo (17), both (6)	Ipi, 8.6 (1-year 41.2%) Nivo,12 (1-year 63%)	NR	NR	NR
*Patel* et al.*, 2017* [29]	20	Ipi plus SRS (whitin 4 months)	8 (1-year 37.1%)	1-year LC and DBF 71 and 12%	NR	18% at 1 year
Skrepnik et al., 2017 [30]	25	Ipi before or concurrent (within 1 month)	35 (1-year and 2-year 83 and 64%)	16.7 (1-year and 2-year 52 and 34.8%)	NR	20.7 5% symptomatic
*Chen* et al.*, 2018* [31]	23 (28)°	concurrent SRS-SRT and Ipi or Pembro	24.7 (1-year 75%)	1-year LC 88%	Grade 2, 42% Grade 3, 0%	27% of 22 metastases confirmed by histology
	12 (51)°	Nonconcurrent SRS-SRT and Ipi or Pembro	14.5 (1-year 53%)	1-year LC 79%	Grade 2, 35% Grade 3, 33%	
*Nardin* et al.*, 2018* [32]	25	SRS and Pembro	15.3	8.4 (6-months LC 80%)	NR	6.8%
Current series	45	concurrent SRS-SRT and Nivo (within 1 week)	22 (1-year 78%)	1-year 42% 1-year LC and DBC 85 and 46%	Grade 3, 11%	25% at 1-year
	35	Concurrent SRS-SRT and Ipi (within 1 week)	14.7 (1-year 68%)	1-year 17% 1-year LC and DBC 70 and 20%	Grade 3, 6%	17% at 1 year

*Ipi* Ipilimumab, *Nivo* Nivolumab, *Pembro* Pembrolizumab, *SRS* Stereotactic radiosurgery, *SRT* Stereotactic radiotherapy, *LC* local control, *DBC* distant brain control, *NR* not reported; ° Study including patients with brain metastases from melanoma, non small-cell lung cancer, and renal cell carcinoma

Several factors had a positive impact on patient outcomes. An intriguing finding of our study was the significantly better intracranial and extracranial PFS at 6 and 12 months in patients receiving multi-fraction SRS and concurrent checkpoint inhibitors, either nivolumab or ipilimumab. Emerging evidence suggests that radiotherapy and immunotherapy may have synergistic effects [35–44]. Preclinical studies have shown that the combination of radiotherapy and targeted PD-1/PD-L1 therapy activates cytotoxic T-cells, reduces myeloid-derived suppressor cells, and may induce an abscopal response, as defined by a significant growth inhibition of the tumor outside the irradiated field [35–37]. Similarly, several preclinical and clinical studies have reported the enhanced immunostimulatory effects of radiotherapy when given in combination with anti-CTL-4 antibodies for either irradiated or non-irradiated tumors [38–44]**.** Dewan et al. [39] demonstrated that an abscopal effect occurred only in mice treated with anti-CTLA-4 antibodies combined to multi-fraction SRS (3 x 8 Gy), but not to single-fraction SRS (20 Gy). Consistent with this finding, abscopal responses have been reported in patients receiving hypofractionated radiotherapy and ipilimumab [40–44].

Although our results support the synergistic effects between multi-fraction SRS and either ipilimumab or nivolumab, large prospective studies are required to confirm our findings. Currently, there are no prospective controlled data showing that adding radiotherapy, either SRS or fractionated radiotherapy, to PD-1/PD-L1 or CTLA-4 inhibition may enhance abscopal responses. The question whether the combination of checkpoint inhibition and radiotherapy improves the efficacy of checkpoint inhibition alone in different tumors is being addressed in ongoing clinical trials [45]**.**

The management paradigm of melanoma brain metastases is rapidly changing. Both PD-1/PD-L1 or CTLA-4 checkpoint inhibitors have shown activity in patients with melanoma brain metastases, with a response rate of up to one third of patients [9–11]. More recently, a combination of checkpoint inhibitors has been explored as a new strategy to improve the outcome over monotherapy. Two prospective trials assessing the efficacy and safety of combining nivolumab and ipilimumab in patients with asymptomatic melanoma brain metastases have showed durable intracranial response in about 65% of patients [11, 12]; however, grade 3 or 4 occurred in more than 50% of patients causing interruption of treatment in up to 26% of patients. Even though toxicity of combined checkpoint inhibitors occurs in a significant proportion of patients, systemic therapy alone may represent a reasonable initial approach for asymptomatic brain metastases; however, its efficacy in symptomatic lesions remains to be proven. In absence of controlled randomized trials, results observed in our study suggest that combined immunotherapy and SRS should be considered in the setting of large symptomatic melanoma brain metastases. Notably, efficacy of treatments was apparently maintained in patients receiving corticosteroids, for whom the response to immunotherapy alone seems to be less effective [9, 46].

Treatments were generally well tolerated. Early or late radiological changes suggestive of RN were shown in one third of patients, with grade 3 neurotoxicity occurring in 9% of them. The risk was consistent with those observed in other series of concurrent checkpoint inhibitors and SRS [24, 26, 27, 29, 30]. Notably, most of radiological changes occurred in the first 3-4 months after SRS and were typically characterized by an enlargement of enhanced lesions and increased perilesional edema, so called pseudoprogression. Radiological findings resolved in 6-8 weeks in the majority of patients and were rarely associated with neurological symptoms; however, a strict follow-up imaging is recommended in these patients for distinguishing pseudoprogression from true tumor progression. Even though the risk of RN after concurrent therapy is similar to that observed with SRS alone [14, 47, 48], the absence of RN after combined ipilimumab and nivolumab [11, 12] addresses important questions about the optimal treatment strategy for patients with melanoma brain metastases. The use of SRS as up-front or salvage therapy to maximize benefit and minimize toxicity needs to be explored in future trials.

The current study has several limitations, owing to its retrospective nature. The presence of unmeasured baseline characteristics, such as presence of comorbidities, levels of PD-1/PD-L1 expression, extension of extracranial disease, and previous systemic treatments is likely to introduce selection bias. Moreover, different doses and duration of corticosteroids for controlling neurological symptoms, or different salvage therapies at progression may contribute to the observed differences in clinical outcomes between groups. Nevertheless, our results demonstrate that concurrent SRS and nivolumab or ipilimumab is associated with high intracranial activity.

In conclusion, our study shows that SRS combined with nivolumab provides better intracranial control than SRS and ipilimumab in patients with both symptomatic and asymptomatic melanoma brain metastases, although a significant subset of patients receiving immunotherapy and concurrent SRS may develop symptomatic RN. Combination of nivolumab with multi-fraction SRS has the potential to provide a strong synergistic effect. The efficacy and safety of different radiation schedules and checkpoint inhibitors over other therapeutic strategies require further investigation.

## Additional files

Additional file1: **Figure S1.** Kaplan-Meier analysis of extracranial progression-free survival (S1,A) and global progression-free survival (S1,B) for patients receiving SRS and ipilimumab (blue line) or nivolumab (red line). 6-month and 1-year extracranial PFS rates were 57 and 37% and 42 and 17%, respectively, in SRS and ipilimumab or nivolumab group. Respective 6-month and 1-year global PFS rates were 53 and 36% and 34 and 17%. (PDF 255 kb)

Additional file 2: **Figure S2.** Kaplan-Meier analysis of intracranial progression-free survival (PFS) and overall survival (OS) in patients who received dexamethasone (yes, blue line) or not (not, red line) during treatments. For PFS, 6-month and 12-month rates were 42.3 and 24.2%, and 73.6 and 35.9% respectively, in patients receiving dexamethasone or not. For OS, respective rates were 91.2 and 57.3% and 96 and 76.1%. (PDF 40 kb)

## Abbreviations

BRAF: Serine/threonine-protein kinase B-Raf
CNS: Central nervous system
CR: Complete response
CT: Computed tomography
CTLA-4: Cytotoxic T-lymphocyte antigen-4
CTV: Clinical target volume
DBC: Distant brain control
DS-GPA: Diagnosis-Specific graded prognostic assessment
GTV: Gross tumor volume
LC: Local control
MEK: Mitogen-activated protein kinase kinase
MRI: Magnetic resonance imaging
OS: Overall survival
PD: Progressive disease
PD-1: Programmed cell death 1
PD-L1: Programmed cell death-ligands 1
PET: Positron emission tomography
PFS: Progression-free survival
PR: Partial response
PTV: Planning target volume
RN: Radiation-induced Brain Necrosis
SD: Stable disease
SRS: Stereotactic radiosurgery
WBRT: Whole brain radiation therapy
