# Supplementary figures and images for: Stereotactic radiosurgery combined with nivolumab or Ipilimumab for patients with melanoma brain metastases: evaluation of brain control and toxicity
Source: J Immunother Cancer. 2019 Apr 11;7:102. doi: 10.1186/s40425-019-0588-y (PMC6458744; doi:10.1186/s40425-019-0588-y)

Figure S1

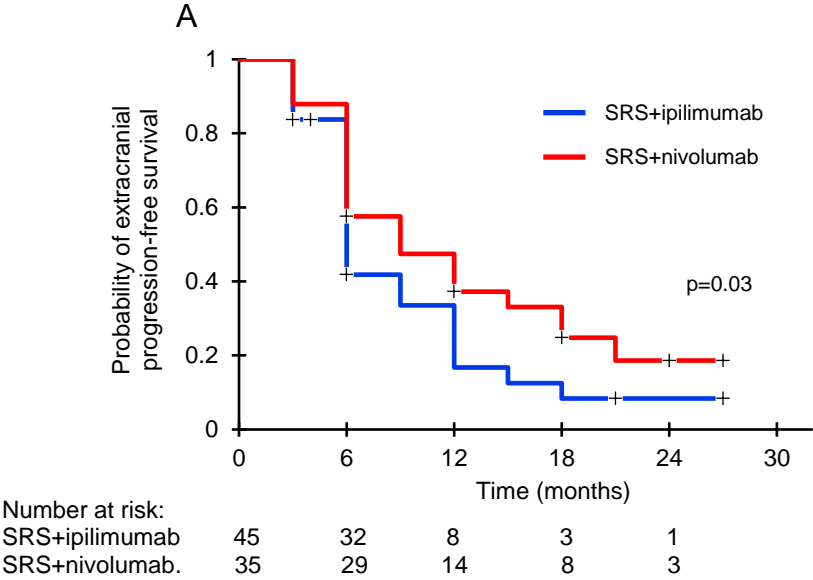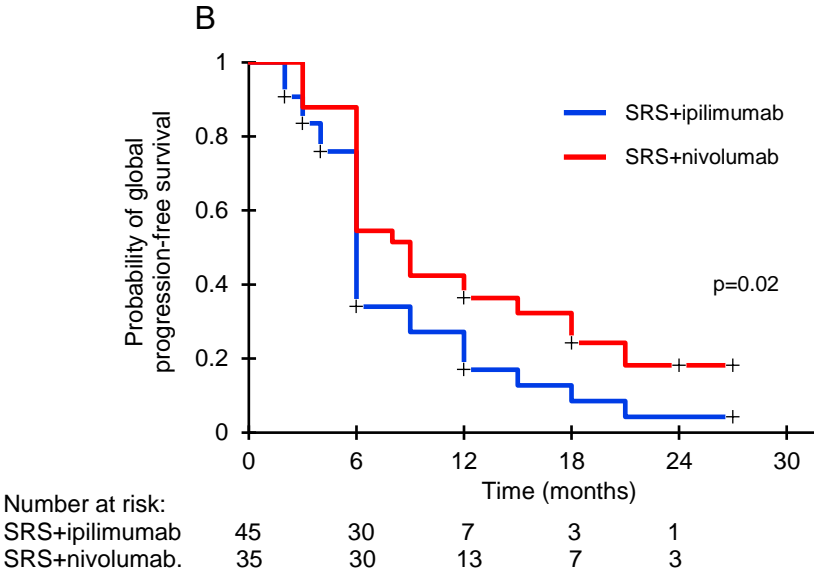

Supplement: Supplementary file 1 — Figure S1. Kaplan-Meier analysis of extracranial progression-free survival (S1,A) and global progression-free survival (S1,B) for patients receiving SRS and ipilimumab (blue line) or nivolumab (red line). 6-month and 1-year extracranial PFS rates were 57 and 37% and 42 and 17%, respectively, in SRS and ipilimumab or nivolumab group. Respective 6-month and 1-year global PFS rates were 53 and 36% and 34 and 17%. (PDF 255 kb) [file 40425_2019_588_MOESM1_ESM.pdf]

Figure S2

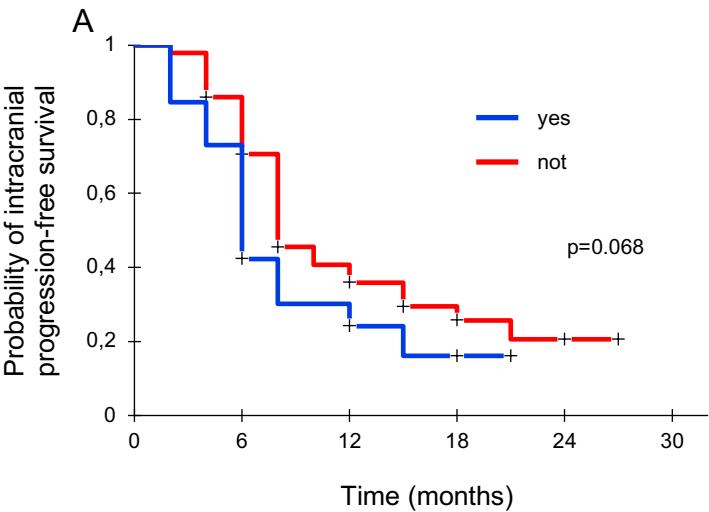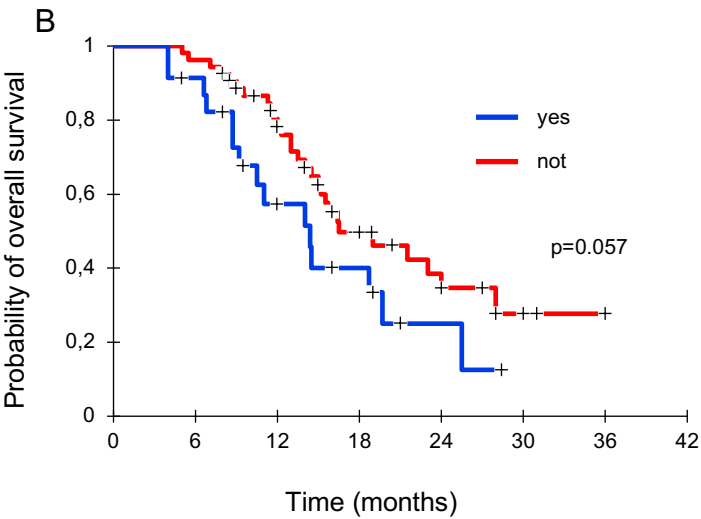

Supplement: Supplementary file 2 — Figure S2. Kaplan-Meier analysis of intracranial progression-free survival (PFS) and overall survival (OS) in patients who received dexamethasone (yes, blue line) or not (not, red line) during treatments. For PFS, 6-month and 12-month rates were 42.3 and 24.2%, and 73.6 and 35.9% respectively, in patients receiving dexamethasone or not. For OS, respective rates were 91.2 and 57.3% and 96 and 76.1%. (PDF 40 kb) [file 40425_2019_588_MOESM2_ESM.pdf]
